# Clinical Outcomes of COVID-19-Induced Acute Respiratory Distress Syndrome in Patients With Three Different Respiratory Support Modalities: A Retrospective Cohort Study

**DOI:** 10.7759/cureus.31991

**Published:** 2022-11-28

**Authors:** Ans Alamami, Tahir Imaduddeen, Ezzedi A Ibrahim, Abdusalam S Ibrahim, Tasleem Raza

**Affiliations:** 1 Critical Care Medicine, Hamad Medical Corporation, Doha, QAT; 2 Critical Care Medicine, Sunnybrook Health Sciences Centre, Toronto, CAN

**Keywords:** niv, p-sili, ards, sars-cov-2, covid-19

## Abstract

Introduction

Severe acute respiratory syndrome coronavirus 2 (SARS-CoV-2)-induced acute respiratory failure and acute respiratory distress syndrome (ARDS) had a considerable impact on intensive care utilization and resource optimization. Multiple modalities for respiratory support were implemented during the COVID-19 pandemic with the main concern of being able to identify those patients at high risk of rapidly progressive respiratory failure, whom the early initiation of invasive respiratory support would, in particular, affect the outcome in comparison to the noninvasive management strategy.

Objectives

In our cohort study, we describe demographic characteristics, respiratory support modalities, and their relation to patient outcomes.

Method

Patients 18 years of age and older who were admitted to a tertiary center COVID-19-dedicated medical intensive care unit (MICU) in Qatar between March 2020 and May 2020 with a confirmed diagnosis of COVID-19 pneumonia were included in this study. Patients were divided into invasive or noninvasive, and those who required invasive strategy were subdivided into the early intubation group (patients who were intubated within 72 hours of intensive care unit (ICU) admission) and the late intubation group (patients who were intubated after 72 hours from ICU admission). The primary outcome was ICU and hospital mortality, and the secondary effects were the length of stay and mortality determinants.

Results

A total of 686 patients were admitted to the medical intensive care unit (MICU) during the study period. There were 222 (32.4%), 131 (19.1%), and 333 (48.5%) patients in the early, late, and not intubated groups, respectively. Compared to the late intubated group, the early intubated group had a higher proportion of males. Diabetes (39.8%) was the most common comorbidity, followed by hypertension (HTN) (36%) and heart disease (9.8%). The 30-day ICU and hospital mortality were significantly higher in the late intubated group compared to the early intubated group (30.5% versus 15.8%, and 30% versus 16.2%). The median ICU and hospital stay days in the total sample were 8 (interquartile range (IQR): 5-14) and 19 (IQR: 14-25), respectively. The mean estimates of 30-day ICU survival times for early intubated, late intubated, and not intubated groups were 25.14 (95% confidence interval (CI): 23.71, 26.57), 23.35 (95% CI: 21.63, 25.07), and 29.91 (95% CI: 29.74, 30.09) respectively.

Conclusions

In our study, the COVID-19 ARDS patients who required early invasive ventilatory support and in whom the physiological parameter was more severe (Acute Physiology and Chronic Health Evaluation II (APACHE II) and Sequential Organ Failure Assessment (SOFA) scores) had a better outcome than the late intubation group. Age more than 60 years old, diabetes, hypertension, chronic kidney disease (CKD), and chronic liver disease (CLD) were the main predictor of mortality in total.

## Introduction

Severe acute respiratory syndrome coronavirus 2 (SARS-CoV-2) was first identified in December 2019 in Wuhan, China [1]. Since then, it has progressed to become a global pandemic that is still ongoing. Qatar identified its first COVID-19 case on February 27, 2020 [2], and since then has reached more than 500 million cases globally. SARS-CoV-2 has mutated multiple times since early 2020, and different strains have variables leading to a higher risk of severe disease and hospitalization with some strains [3-6]. The basic pathophysiology of severe viral pneumonia is hypoxic respiratory failure secondary to severe acute respiratory distress syndrome (ARDS). During the initial waves of infections, most of the patients (81%) had mild to moderate symptoms, and around 14% developed severe symptoms requiring hospitalization, 5% of whom suffered critical symptoms (respiratory failure, shock, or multiorgan failure) and had a high rate of intensive care unit (ICU) admission and demand for ventilatory support [7]. This has placed a heavy burden on the healthcare system around the world. Earlier studies and observations, including one metanalysis [8], did not reveal a significant difference in mortality comparing early intubation to late intubation strategy in a COVID-19 patient, taking into consideration that most of the early intubated group were defined as patients who required intubation within 24 hours of hospital admission [9,10]. In our cohort, we defined the early intubation group as patients who require intubation within 72 hours of hospital admission to evaluate the mortality outcome and the mortality predictor of early and late intubation groups.

## Materials and methods

Aim of the study

The aim was to study the clinical impact of three treatment modalities (noninvasive ventilation versus early intubation versus late intubation) on patients admitted to a single tertiary COVID-19-dedicated intensive care unit from April 1, 2020, to May 31, 2020, with moderate to severe COVID-19-related pneumonia/acute respiratory distress syndrome in Doha, Qatar. Patients will be divided into three arms and matched with baseline characteristics. One arm will include patients who were given invasive ventilatory support within 72 hours (early) of admission, another arm those who were given noninvasive ventilatory (NIV) support exclusively (noninvasive positive pressure ventilation (NIPPV), high-flow nasal cannula (HFNC), and non-rebreathing mask (NRBM) ventilation), and another those who were intubated after 72 hours for failed NIV support (late).

Study design and setting

This is a retrospective observational study reviewing the data registry of patients admitted to the medical intensive care unit (MICU) in three MICUs across Hamad Medical Corporation in Qatar due to COVID-19. The intensive care unit (ICU) centers involved are within a single national critical care network in Qatar. A data collector collects the data on a specific form prepared by the study investigator. The study was approved by the Hamad Medical Corporation Medical Research Council (MRC-05-236) under an expedited process, with a waiver for consent to participate.

Inclusion criteria

Patients aged ≥18 and <65, those with confirmed SARS-CoV-2 by polymerase chain reaction (PCR), and those with acute respiratory failure requiring >10 L of oxygen supply or who were intubated were included in the present study.

Exclusion criteria

Patients were excluded if they are less than 18 years old and/or pregnant and if there is a contraindication to noninvasive ventilation initiation (decreased level of consciousness and vomiting).

Data collection

Patient data were extracted from electronic health records (Cerner Millennium). We used this registry from April 1, 2020, to May 31, 2020. Data were placed in a specific data collection sheet coded for patient privacy with access restricted to the study investigator.

Variables of interest

The variables of interest are as follows: age, gender, duration, types of oxygen supplementation (whether through a face mask, high-flow nasal cannula, or noninvasive ventilation (NIV)), and timing of intubation. The Acute Physiology and Chronic Health Evaluation (APACHE II) score, Sequential Organ Failure Assessment (SOFA) scores, and partial pressure of oxygen (PO2)/fraction of inspired oxygen (FIO2) ratio were calculated from the electronic medical record (EMR) based on the closest value to the date of ICU admission.

Study comparison and outcomes

The primary exposure was the timing of tracheal intubation after the ICU admission. Based on that, the included patients of interest were categorized into three groups; the first group included patients who were intubated and ventilated in less than 72 hours from the ICU admission, the second group were patients intubated after 72 hours from hospital admission, and the last group was patients who did not undergo tracheal intubation during their hospital course.

The primary outcome was the 30-day ICU and hospital mortality of each group. The secondary outcomes were the duration of ICU stay, hospital stay, and predictors of poor prognosis.

All patients were treated with a single unified treatment protocol, which did not include remdesivir (as the evidence was not established at the time of the study), and all of the included subjects were treated in the same manner.

Statistical analysis

Firstly, we summarized descriptive statistics of the sample using the mean and standard deviation (SD) or median and interquartile range (IQR) for continuous variables and frequencies for categorical variables. We stratified the sample based on the timing of the intubation (early, late, and not intubated). We compared the characteristics of the stratified samples (early versus late intubated and early versus not intubated) using the independent samples t-test or Mann-Whitney U test for continuous variables and χ2 test or Fisher’s exact test for categorical variables. Further, we compared the characteristics of the main sample according to the 30-day ICU and hospital mortality (dead versus alive) using the same statistical tests.

We calculated 30-day mortality after ICU admission and hospital admission separately. Days from ICU/hospital admission to death (event) or 30 days (censoring) were used for the survival analysis. At censoring, a patient might be alive in the ICU/hospital or alive and discharged. We plotted the survival curves until 30 days following the ICU and hospital admissions separately using Kaplan-Meier survival estimates and compared the groups (early versus late intubated and early versus not intubated) using the log-rank test. Next, we explored the association of intubation (exposure) and time to 30-day ICU or hospital mortality (outcome) in the total sample using multivariable Cox proportional hazards regression models. The risk factors/covariables were defined a priori based on the current evidence. Similarly, we assessed the effect of intubation time (early versus late intubated and early versus not intubated) and time to 30-day ICU or hospital mortality in the respective stratified groups. We reported results using hazard ratios (HRs) and 95% confidence intervals (CIs).

In the secondary analysis, we used multivariable generalized linear regression models to investigate the associations of intubation and baseline characteristics with the ICU or hospital stay days. We analyzed two steps. First, we assessed the association in all patients without considering whether they were dead or alive. We included ICU mortality for ICU stay days and hospital mortality for hospital stay days as predictors in the first model. Next, we conducted a sensitivity analysis on patients alive or alive and discharged during the study period. Further, we extended our analysis to subsamples (early, late, and not intubated groups). We reported results using beta values and 95% confidence intervals.

Further, we supplemented the main results of multivariable analysis using sensitivity analysis on imputed datasets. Missing values in the baseline risk factors/covariates were imputed using the fully conditional specification method of multiple imputations. However, we did not impute independent or dependent variables. We reported pooled estimates of 10 imputed datasets.

We analyzed data using Statistical Package for the Social Sciences (SPSS) version 25.0 (IBM SPSS Statistics, Armonk, NY, USA).

## Results

There were 686 patients admitted to the MICU during the study period. There were 222 (32.4%), 131 (19.1%), and 333 (48.5%) patients in early, late, and not intubated groups, respectively. The descriptive statistics of the total and stratified samples (early, late, and not intubated groups) are summarized in Table 1.

**Table 1:** Descriptive statistics and comparisons of groups stratified according to intubation and the timing of intubation In the early intubated group, there were 1, 26, 9, 9, 2, 159, 212, 197, 25, 120, and 215 missing values in ICU stay days, hospital stay days, APACHE II, SOFA score, mechanical ventilation days, NRBM time, HFNC time, NIV time, hospital mortality, number of sessions, and ECMO. In the late intubated group, there were 1, 12, 1, 13, 13, 5, 50, 123, 103, 11, 66, and 120 in ICU stay days, hospital stay days, PO2/FIO2 ratio, APACHE II, SOFA score, mechanical ventilation days, NRBM time, HFNC time, NIV time, hospital mortality, number of sessions, and ECMO. In the not intubated group, there were 7, 25, 24, 24, 165, 315, 259, 1, 7, and 228 in hospital stay days, APACHE II, SOFA score, NRBM time, HFNC time, NIV time, ICU mortality, hospital mortality, and the number of sessions. ^a^p-values were derived from independent samples t-test or Mann-Whitney U test for continuous variables and χ2 test or Fisher’s exact test for categorical variables. ^b^Applicable only in intubated patients. SD: standard deviation; IQR: interquartile range; N/A: not applicable; ICU: intensive care unit; APACHE II: Acute Physiology and Chronic Health Evaluation II; SOFA: Sequential Organ Failure Assessment; NRBM: non-rebreather mask; HFNC: high-flow nasal cannula, NIV: noninvasive ventilation, ECMO: extracorporeal membrane oxygenation

Patient characteristics	Number	Total	Early intubated	Late intubated	Not intubated	Early versus late intubated	Early versus not intubated
		(n = 686)	(n = 222)	(n= 131)	(n = 333)	p-value^a^	p-value^a^
Age, mean (SD)	686	49.05 (13.12)	49.09 (12.99)	53.54 (13.38)	47.26 (12.71)	<0.01	0.1
Sex, male, number (%)	686	609 (88.8)	206 (92.8)	106 (80.9)	297 (89.2)	<0.01	0.18
Comorbidities, yes, number (%)							
Hypertension	686	247 (36)	85 (38.3)	55 (42)	107 (32.1)	0.5	0.15
Diabetes	686	273 (39.8)	89 (40.1)	66 (50.4)	118 (35.4)	0.08	0.28
Kidney disease	686	37 (5.4)	15 (6.8)	8 (6.1)	14 (4.2)	1	0.24
Kidney disease on dialysis	686	10 (1.5)	2 (0.9)	2 (1.5)	6 (1.8)	0.63	0.49
Liver disease	686	3 (0.4)	1 (0.5)	1 (0.8)	1 (0.3)	1	1
Heart disease	686	67 (9.8)	20 (9)	20 (15.3)	27 (8.1)	0.08	0.76
Lung disease	686	39 (5.7)	8 (3.6)	13 (9.9)	18 (5.4)	0.02	0.41
Immunosuppression	686	19 (2.8)	5 (2.3)	7 (5.3)	7 (2.1)	0.14	1
Metastatic cancer	686	9 (1.3)	2 (0.9)	2 (1.5)	5 (1.5)	0.63	0.71
Hematological malignancy	686	8 (1.2)	3 (1.4)	5 (3.8)	0 (0)	0.15	0.06
PO2/FIO2 ratio, median (IQR)	660	106 (81-141)	111 (82-146)	96 (76-128)	104 (82-154)	0.01	0.79
APACHE II, median (IQR)	640	12 (9-16)	14 (10-18)	13 (10-18)	10 (8-14)	0.68	<0.01
SOFA score, median (IQR)	640	3 (2-5)	5 (3-7)	4 (3-5)	2 (2-3)	0.02	<0.01
NRBM, yes, number (%)	686	518 (75.5)	149 (67.1)	120 (91.6)	249 (74.8)	<0.01	0.06
NRBM time, median (IQR)	312	32 (11-77)	10 (4-25)	40 (16-99)	41 (17-85)	<0.01	<0.01
HFNC, yes, number (%)	686	55 (8)	18 (8.1)	12 (9.2)	25 (7.5)	0.84	0.87
HFNC time, median (IQR)	36	30 (0-78)	0 (0-16)	40 (20-129)	53 (0-109)	<0.01	0.01
NIV, yes, number (%)	686	158 (23)	43 (19.4)	35 (26.7)	80 (24)	0.11	0.21
NIV time, median (IQR)	127	14 (5-32)	6 (3-18)	26 (5-70)	16 (8-30)	0.01	0.01
ICU 30-day mortality, dead, number (%)	685	76 (11.1)	35 (15.8)	40 (30.5)	1 (0.3)	<0.01	<0.01
Hospital 30-day mortality, dead, number (%)	643	69 (10.7)	32 (16.2)	36 (30)	1 (0.3)	<0.01	<0.01
ICU stay days, median (IQR)	684	8 (5-14)	11 (8-18)	14 (9-23)	6 (4-8)	<0.01	<0.01
Hospital stay days, median (IQR)	641	19 (14-25)	20 (15-27)	24 (18-34)	17 (13-22)	<0.01	<0.01
Readmission, yes, number (%)	686	20 (2.9)	9 (4.1)	7 (5.3)	4 (1.2)	0.6	0.04
Prone position, yes, number (%)	686	232 (33.8)	92 (41.4)	56 (42.7)	84 (25.2)	0.82	<0.01
Number of sessions, number (%)	272						
1		174 (64)	58 (56.9)	34 (52.3)	82 (78.1)	0.82	<0.01
2		74 (27.2)	31 (30.4)	21 (32.3)	22 (21)		
3 or more		24 (8.8)	13 (12.7)	10 (15.4)	1 (1)		
Extubation, yes, number (%)^b^	353	261 (73.9)	176 (79.3)	85 (64.9)	N/A	<0.01	-
Reintubation, yes, number (%)^b^	353	24 (6.8)	15 (6.8)	9 (6.9)	N/A	1	-
Tracheostomy, yes, number (%)^b^	353	13 (3.7)	6 (2.7)	7 (5.3)	N/A	0.24	-
Mechanical ventilation days							
Median (IQR)^b^	346	6 (4-12)	6 (4-11)	6 (3-13)	N/A	0.64	-
ECMO, yes, number (%)^b^	18	16 (88.9)	6 (85.7)	10 (90.9)	N/A	1	-

Patients were on average 49 (SD = 13.12) years old, and 89% were men. Patients in the late intubated group were significantly older (54 versus 49 years) compared to the early intubated group. The early intubated group had a higher proportion of males compared to the late intubated group. Diabetes (39.8%) was the most common comorbidity, followed by hypertension (HTN) (36%) and heart disease (9.8%). However, there was no significant difference in comorbidities between the comparison groups except for a higher proportion of lung disease in the late intubated group compared to the early intubated group. The early intubated group had a significantly higher PO2/FIO2 ratio and SOFA score compared to the late intubated group and higher APACHE II and SOFA scores compared to the not intubated group. The early intubated group had higher readmission, prone position, and several sessions compared to the not intubated group. Further, the late intubated group had a higher extubation rate compared to the early intubated group, but reintubation, tracheostomy rates, and mechanical ventilation days were similar.

The median days from hospital admission to ICU admission were one (IQR: 1-2), three (IQR: 2-5), and two (IQR: 1-5) days for early, late, and not intubated groups, respectively. Further, the median days from hospital admission to intubation were one (IQR: 0-2) and six (IQR: 4-9) for early and late intubated groups, respectively. The 30-day ICU and hospital mortality were 11.1% and 10.7%, respectively, in the total group (Table 2).

**Table 2:** Clinical outcomes and comparisons of groups stratified according to intubation and the timing of intubation ^a^p-values were derived from independent samples t-test or Mann-Whitney U test for continuous variables and χ2 test or Fisher’s exact test for categorical variables. ^b^Applicable only in intubated patients. ICU: intensive care unit; IQR: interquartile range; N/A: not applicable

Patient characteristics	Number	Total	Early intubated	Late intubated	Not intubated	Early versus late intubated	Early versus not intubated
		(n = 686)	(n = 222 )	(n = 131)	(n = 333)	p-value^a^	p-value^a^
ICU 30-day mortality, dead, number (%)	685	76 (11.1)	35 (15.8)	40 (30.5)	1 (0.3)	<0.01	<0.01
Hospital 30-day mortality, dead, number (%)	643	69 (10.7)	32 (16.2)	36 (30)	1 (0.3)	<0.01	<0.01
ICU stay days, median (IQR)	684	8 (5-14)	11 (8-18)	14 (9-23)	6 (4-8)	<0.01	<0.01
Hospital stay days, median (IQR)	641	19 (14-25)	20 (15-27)	24 (18-34)	17 (13-22)	<0.01	<0.01
Readmission, yes, number (%)	686	20 (2.9)	9 (4.1)	7 (5.3)	4 (1.2)	0.6	0.04
Prone position, yes, number (%)	686	232 (33.8)	92 (41.4)	56 (42.7)	84 (25.2)	0.82	<0.01
Number of sessions, number (%)	272						
1		174 (64)	58 (56.9)	34 (52.3)	82 (78.1)	0.82	<0.01
2		74 (27.2)	31 (30.4)	21 (32.3)	22 (21)		
3 or more		24 (8.8)	13 (12.7)	10 (15.4)	1 (1)		
Extubation, yes, number (%)^b^	353	261 (73.9)	176 (79.3)	85 (64.9)	N/A	<0.01	-
Reintubation, yes, number (%)^b^	353	24 (6.8)	15 (6.8)	9 (6.9)	N/A	1	-
Tracheostomy, yes, number (%)^b^	353	13 (3.7)	6 (2.7)	7 (5.3)	N/A	0.24	-
Mechanical ventilation days							
Median (IQR)^b^	346	6 (4-12)	6 (4-11)	6 (3-13)	N/A	0.64	-
ECMO, yes, number (%)^b^	18	16 (88.9)	6 (85.7)	10 (90.9)	N/A	1	-

Interestingly, the 30-day ICU and hospital mortality were significantly higher in the late intubated group compared to the early intubated group (30.5% versus 15.8% and 30% versus 16.2%). There was only one (0.3%) patient with mortality in the not intubated group. Thus, 30-day ICU and hospital mortality were higher in the early intubated group compared to the not intubated group. The median ICU and hospital stay days in the total sample were eight (IQR: 5-14) and 19 (IQR: 14-25), respectively. The ICU and hospital stay days were significantly higher in the late intubated compared to the early intubated group and in the early intubated compared to the not intubated group. The descriptive statistics stratified by 30-day ICU and hospital mortality status are summarized in Table 3.

**Table 3:** Descriptive statistics and comparisons of groups according to their 30-day mortality status ^a^p-values were derived from independent samples t-test or Mann-Whitney U test for continuous variables and χ2 test or Fisher’s exact test for categorical variables. ^b^Applicable only in intubated patients. SD: standard deviation; IQR: interquartile range; N/A: not applicable; ICU: intensive care unit; APACHE II: Acute Physiology and Chronic Health Evaluation II; SOFA: Sequential Organ Failure Assessment; NRBM: non-rebreather mask; HFNC: high-flow nasal cannula, NIV: noninvasive ventilation, ECMO: extracorporeal membrane oxygenation

Patient characteristics	Number	30-day ICU mortality			Number	30-day hospital mortality		
		Dead	Alive	p-value^a^		Dead	Alive	p-value^a^
		(n = 76)	(n = 609)			(n = 69)	(n = 574)	
Age, mean (SD)	685	59.50 (15.06)	47.67 (12.12)	<0.01	643	59.30 (15.61)	47.59 (12.00)	<0.01
Sex, male, number (%)	685	63 (82.9)	545 (89.5)	0.12	643	59 (85.5)	515 (89.7)	0.3
Comorbidities, yes, number (%)								
Hypertension	685	46 (60.5)	200 (32.8)	<0.01	643	41 (59.4)	192 (33.4)	<0.01
Diabetes	685	48 (63.2)	225 (36.9)	<0.01	643	43 (62.3)	213 (37.1)	<0.01
Kidney disease	685	8 (10.5)	28 (4.6)	0.05	643	7 (10.1)	24 (4.2)	0.04
Kidney disease on dialysis	685	2 (2.6)	8 (1.3)	0.31	643	2 (2.9)	7 (1.2)	0.25
Liver disease	685	2 (2.6)	1 (0.2)	0.03	643	1 (1.4)	2 (0.3)	0.29
Heart disease	685	18 (23.7)	48 (7.9)	<0.01	643	16 (23.2)	46 (8)	<0.01
Lung disease	685	5 (6.6)	34 (5.6)	0.79	643	4 (5.8)	33 (5.7)	1
Immunosuppression	685	9 (11.8)	10 (1.6)	<0.01	643	8 (11.6)	11 (1.9)	<0.01
Metastatic cancer	685	4 (5.3)	5 (0.8)	0.01	643	3 (4.3)	6 (1)	0.06
Hematological malignancy	685	6 (7.9)	2 (0.3)	<0.01	643	5 (7.2)	3 (0.5)	<0.01
PO2/FIO2 ratio, median (IQR)	659	94 (76-129)	107 (82-148)	0.04	620	95 (77-128)	107 (82-150)	0.05
APACHE II, median (IQR)	639	15 (10-21)	12 (9-15)	<0.01	600	15 (10-21)	11 (9-15)	<0.01
SOFA score, median (IQR)	639	5 (3-7)	3 (2-5)	<0.01	600	5 (3-6)	3 (2-5)	<0.01
NRBM, yes, number (%)	685	59 (77.6)	458 (75.2)	0.78	643	52 (75.4)	438 (76.3)	0.88
NRBM time, median (IQR)	312	38 (8-106)	32 (12-73)	0.6	295	38 (11-101)	32 (12-77)	0.8
HFNC, yes, number (%)	685	9 (11.8)	46 (7.6)	0.18	643	8 (11.6)	45 (7.8)	0.26
HFNC time, median (IQR)	36	11 (0-23)	35 (0-84)	0.21	36	16 (0-25)	34 (0-82)	0.29
NIV, yes, number (%)	685	17 (22.4)	141 (23.2)	1	643	16 (23.2)	133 (23.2)	1
NIV time, median (IQR)	127	18 (12-23)	19 (15-26)	0.39	122	24 (4-52)	13 (5-30)	0.33
Intubation, yes, number (%)	685	75 (98.7)	278 (45.6)	<0.01	643	68 (98.6)	249 (43.4)	<0.01
ICU stay days, median (IQR)	684	14 (9-18)	8 (5-13)	<0.01	641	14 (9-17)	8 (5-12)	<0.01
Hospital stay days, median (IQR)	641	19 (14-25)	20 (15-27)	0.01	640	17 (11-21)	8 (5-12)	<0.01
Readmission, yes, number (%)	685	3 (3.9)	17 (2.8)	0.48	643	3 (4.3)	11 (1.9)	0.18
Prone position, yes, number (%)	685	32 (42.1)	200 (32.8)	0.12	643	28 (40.6)	183 (31.9)	0.17
Number of sessions, number (%)	272			<0.01	247			<0.01
1		12 (32.4)	162 (68.9)			11 (34.4)	150 (69.8)	
2		17 (45.9)	57 (24.3)			16 (50)	53 (24.7)	
3 or more		8 (21.6)	16 (6.8)			5 (15.6)	12 (5.6)	
Extubation, yes, number (%)^b^	353	7 (9.3)	254 (91.4)	<0.01	317	5 (7.4)	225 (90.4)	<0.01
Reintubation, yes, number (%)^b^	353	4 (5.3)	20 (7.2)	0.8	317	2 (2.9)	15 (6)	0.54
Tracheostomy, yes, number (%)^b^	353	0 (0)	13 (4.7)	0.08	317	0 (0)	7 (2.8)	0.35
Mechanical ventilation days								
Median (IQR)^b^	346	10 (4-16)	6 (3-10)	0.01	310	9 (4-13)	5 (3-9)	0.01
ECMO, yes, number (%)^b^	18	4 (80)	12 (92.3)	0.49	15	3 (75)	10 (90.9)	0.48

Older patients had high 30-day ICU and hospital mortality. Patients with hypertension, diabetes, liver disease, heart disease, metastatic cancer, hematological malignancy, and immunosuppression medication were associated with higher 30-day ICU and hospital mortality. Further, higher APACHE II and SOFA scores were associated with higher 30-day ICU and hospital mortality, and a lower PO2/FIO2 ratio was only related to 30-day ICU mortality.

30-day ICU and hospital survival times of early, late, and not intubated patients

**Figure 1:**
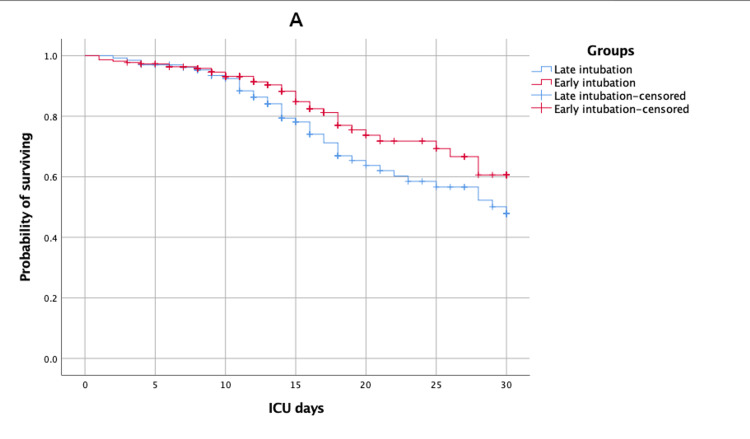
Kaplan-Meier survival estimates during the 30 days following ICU admission according to early versus late intubated group ICU: intensive care unit

**Figure 2:**
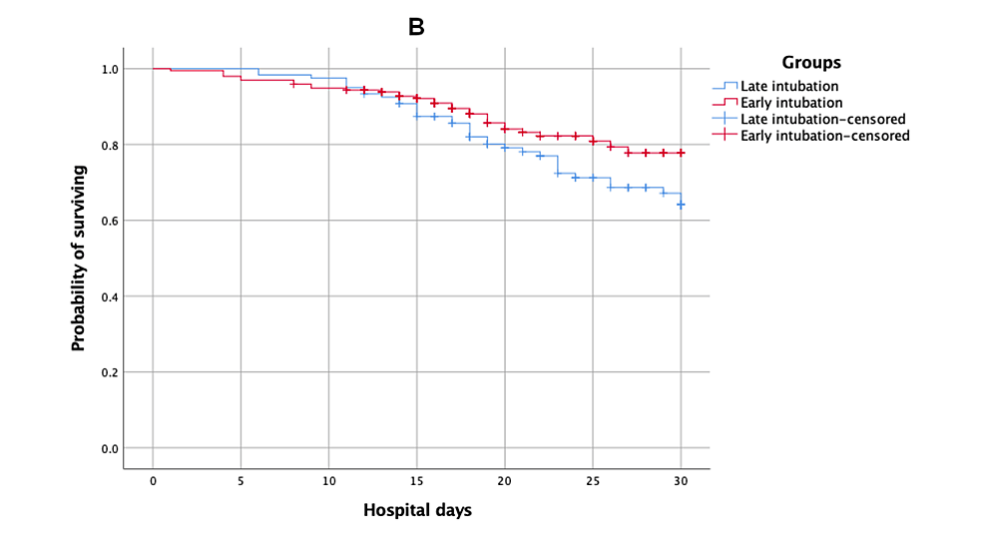
Kaplan-Meier survival estimates during the 30 days following hospital admission according to early versus late intubated group

**Figure 3:**
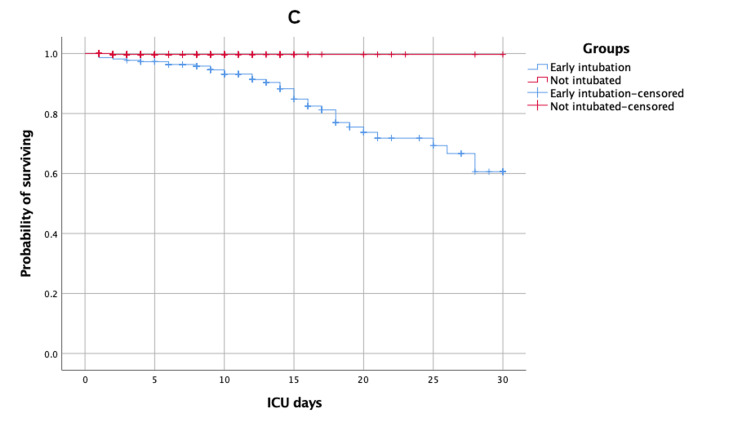
Kaplan-Meier survival estimates during the 30 days following ICU admission according to early versus not intubated group ICU: intensive care unit

**Figure 4:**
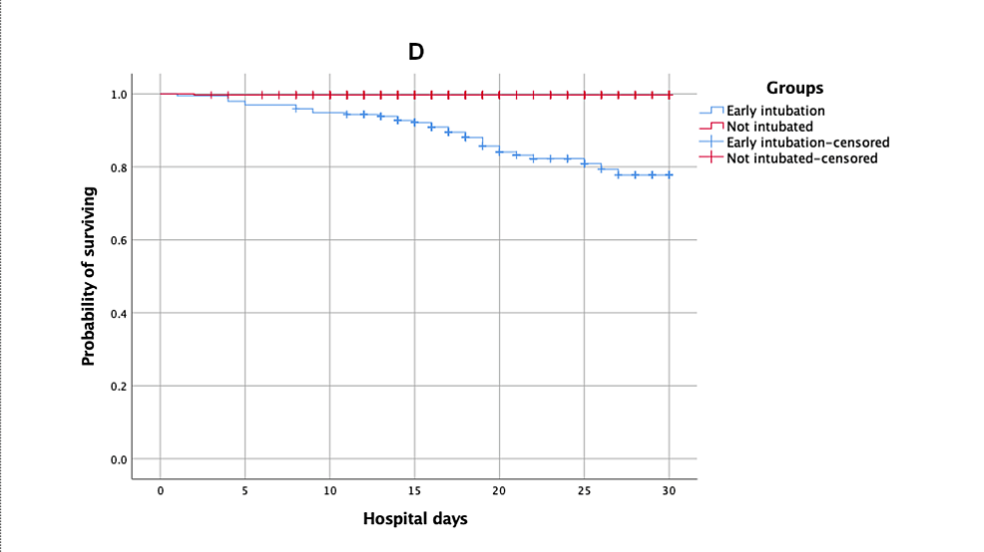
Kaplan-Meier survival estimates during the 30 days following hospital admission according to early versus not intubated group

Kaplan-Meier survival estimates of time to death (survival) after 30 days of ICU and hospital admission for patients of early versus late intubated group and early versus not intubated group are shown in Figures 1-4.

The mean estimates of 30-day ICU survival times for early, late, and not intubated groups were 25.14 (95% CI: 23.71, 26.57), 23.35 (95% CI: 21.63, 25.07), and 29.91 (95% CI: 29.74, 30.09), respectively. There was no significant difference between the early versus late intubated groups (log-rank p-value = 0.10), but there was a significant difference between the early versus not intubated groups (log-rank p-value < 0.01). The mean estimates of 30-day hospital survival times for early, late, and not intubated groups were 27.02 (95% CI: 26.04, 28.01), 26.06 (95% CI: 24.84, 27.28), and 29.91 (95% CI: 29.74, 30.08), respectively. There was no significant difference between the early versus late intubated groups (log-rank p-value = 0.06), but there was a significant difference between the early versus not intubated groups (log-rank p-value < 0.01).

Associations of intubation, time of intubation, and time to 30-day ICU and hospital mortality

Results of multivariable Cox proportional hazards regression in the total, early versus late intubated, and early versus not intubated groups are shown in Table 4.

**Table 4:** Factors associated with time to 30-day ICU and hospital mortality Multivariable analyses included complete cases only. In 30-day ICU mortality, there were 63, 23, and 50 missing values in the total, early versus late intubated, and early versus not intubated groups, respectively. In 30-day hospital mortality, there were 102, 58, and 79 missing values in the total, early versus late intubated, and early versus not intubated groups, respectively. The models were created using the multivariable Cox proportional hazards model. ^a^Exposures with 0 values in some groups. HR: hazard ratio; CI: confidence interval; N/A: not applicable; ref: reference category; ICU: intensive care unit; APACHE II: Acute Physiology and Chronic Health Evaluation II; SOFA: Sequential Organ Failure Assessment

Patient characteristics	30-day ICU mortality			30-day hospital mortality		
	Total sample (n = 623)	Early versus late intubated (n = 330)	Early versus not intubated (n = 505)	Total sample (n = 584)	Early versus late intubated (n = 295)	Early versus not intubated (n = 476)
	HR (95% CI)	HR (95% CI)	HR (95% CI)	HR (95% CI)	HR (95% CI)	HR (95% CI)
Intubation, yes versus no (ref)	16.29 (2.05, 129.47)	N/A	N/A	46.70 (6.05, 360.46)	N/A	N/A
Intubation, early versus late (ref)	N/A	0.83 (0.48, 1.43)	N/A	N/A	0.74 (0.43, 1.29)	N/A
Intubation, early versus no (ref)	N/A	N/A	12.33 (1.38, 109.87)	N/A	N/A	19.99 (2.54, 157.55)
Age in years, one-year increment	1.04 (1.02, 1.07)	1.04 (1.01, 1.06)	1.04 (0.99, 1.07)	1.05 (1.02, 1.08)	1.04 (1.02, 1.07)	1.05 (1.01, 1.09)
Sex, male versus female (ref)	1.14 (0.54, 2.43)	1.17 (0.54, 2.54)	1.99 (0.41, 9.59)	0.96 (0.42, 2.22)	0.97 (0.41, 2.28)	3.57 (0.34, 37.87)
P/F ratio, one-unit increment	1.00 (0.99, 1.01)	1.01 (0.99, 1.01)	1.00 (0.99, 1.01)	1.00 (0.99, 1.00)	1.00 (0.99, 1.00)	1.00 (0.99, 1.01)
APACHE II, one-unit increment	1.01 (0.96, 1.06)	1.01 (0.96, 1.06)	1.02 (0.96,1.09)	1.03 (0.98, 1.08)	1.03 (0.98, 1.08)	1.02 (0.96, 1.08)
SOFA score, one-unit increment	1.01 (0.90, 1.12)	1.00 (0.89, 1.13)	1.04 (0.88, 2.98)	1.02 (0.92, 1.15)	1.03 (0.92, 1.16)	1.13 (0.96, 1.34)
Hypertension, yes versus no (ref)	0.99 (0.51, 1.92)	1.06 (0.54, 2.07)	1.04 (0.37, 2.98)	0.95 (0.50, 1.82)	1.04 (0.54, 2.02)	0.90 (0.33, 2.45)
Diabetes, yes versus no (ref)	1.63 (0.87, 3.05)	1.66 (0.87, 3.19)	2.20 (0.82, 5.90)	1.61 (0.86, 2.99)	1.62 (0.86, 3.07)	1.90 (0.70, 5.10)
Kidney disease, yes versus no (ref)	0.30 (0.10, 0.87)	0.32 (0.11, 0.95)	1.59 (0.27, 9.45)	0.27 (0.08, 0.89)	0.29 (0.09, 0.94)	2.74 (0.37, 20.39)
Kidney disease on dialysis, yes versus no (ref)	3.43 (0.37, 32.01)	3.64 (0.37, 35.81)	9.21 (0.85, 99.36)	4.04 (0.45, 36.34)	5.06 (0.51, 49.80)	4.39 (0.44, 43.89)
Liver disease, yes versus no (ref)	13.61 (2.36, 78.48)	12.51 (2.16, 72.60)	2.36 (0.03, 200.45)	9.65 (1.07, 87.35)	9.24 (1.03, 82.58)	-^a^
Heart disease, yes versus no (ref)	1.81 (0.89, 3.67)	1.69 (0.81, 3.52)	1.02 (0.21, 4.87)	2.11 (1.02, 4.33)	2.07 (0.99, 4.28)	0.53 (0.09, 2.96)
Lung disease, yes versus no (ref)	0.79 (0.18, 3.55)	0.75 (0.16, 3.42)	-^a^	0.80 (0.18, 3.65)	0.76 (0.16, 3.49)	-^a^
Immunosuppression, yes versus no (ref)	4.50 (1.09, 18.64)	3.95 (0.90, 17.40)	3.55 (0.47, 26.73)	6.60 (1.74, 25.13)	6.15 (1.51, 25.01)	5.55 (0.85, 36.24)
Metastatic cancer, yes versus no (ref)	1.66 (0.34, 8.11)	2.12 (0.40, 11.10)	5.22 (0.56, 48.99)	0.79 (0.17, 3.54)	0.90 (0.18, 4.44)	4.24 (0.46, 38.69)
Hematological malignancy, yes versus no (ref)	0.57 (0.12, 2.80)	0.65 (0.13, 3.31)	1.16 (0.09, 15.32)	0.32 (0.06, 1.64)	0.36 (0.07, 1.94)	0.40 (0.03, 5.73)

Intubated patients had higher 30-day ICU and hospital mortality compared to not intubated patients (HR: 16.29, 95% CI: 2.05, 129.47; and HR: 46.70, 95% CI: 6.05, 360.46, respectively). However, the timing of intubation was not associated with 30-day ICU or hospital mortality among intubated patients (early versus late intubated). The increase in age was associated with higher 30-day ICU and hospital mortality in the total group (HR: 1.04, 95% CI: 1.02, 1.07; and HR: 1.05, 95% CI: 1.02, 1.08, respectively). Similarly, increased age was associated with higher 30-day ICU and hospital mortality in the early versus late intubated group (HR: 1.04, 95% CI: 1.01, 1.06; and HR: 1.04, 95% CI: 1.02, 1.07, respectively) and with higher 30-day hospital mortality in early versus not intubated group. The presence of liver disease (HR: 13.61, 95% CI: 2.36, 78.48; and HR: 9.65, 95% CI: 1.07, 87.35) and taking immunosuppression medication (HR: 4.50, 95% CI: 1.09,18.64; and HR: 6.60, 95% CI: 1.74, 25.13) were associated with higher 30-day ICU and hospital mortality, while the presence of kidney disease was associated with lower 30-day ICU and hospital mortality (HR: 0.30, 95% CI: 0.10, 0.87; and HR: 0.27 95% CI: 0.08, 0.89) in the total group. There were similar associations in the early versus late-intubated group except for the absence of an association between taking immunosuppression and 30-day ICU mortality. Further, having heart disease was associated with higher 30-day hospital mortality (HR: 2.11, 95% CI: 1.02, 4.33) in the total group, but we did not observe a significant association in the comparison groups. However, there was no significant association between the baseline characteristics and mortality in the early versus not intubated group except for the positive association between increased age and 30-day hospital mortality.

Factors associated with ICU or hospital stay days

We have summarized the factors associated with ICU or hospital stay days in Tables 5-8. In the model, which included both dead and alive patients, ICU mortality was associated with higher ICU stay days in the total (Beta = 4.20, 95% CI: 2.08, 6.31) and late (Beta = 7.49, 95% CI: 2.13, 12.84) intubated groups (Table 5).

**Table 5:** Factors associated with ICU stay days of the patients (dead or alive) There were 64, 10, 14, and 40 missing values in total, early, late, and not intubated groups. Models were constructed using generalized linear regression with the ICU stay days as the continuous outcome. Betas represent an increase or decrease in the ICU stay days for every unit increase in continuous variables, and ICU mortality, intubation, male sex, the presence of comorbidity compared to the ICU alive, not intubated, female sex, and absence of comorbidity. ^a^Exposures with 0 values in some groups. CI: confidence interval; N/A: not applicable; ref: reference category; ICU: intensive care unit; APACHE II: Acute Physiology and Chronic Health Evaluation II; SOFA: Sequential Organ Failure Assessment

Patient characteristics	Total (n = 622)	Early intubated (n = 212)	Late intubated (n = 117)	Not intubated (n = 293)
	Beta (95% CI)	Beta (95% CI)	Beta (95% CI)	Beta (95% CI)
ICU mortality, yes versus no (ref)	4.20 (2.08, 6.31)	0.26 (-3.35, 3.87)	7.49 (2.13, 12.84)	-6.39 (-14.97, 2.19)
Intubation, yes versus no (ref)	6.78 (5.35, 8.21)	N/A	N/A	N/A
Age (years), one-year increment	0.06 (0.01, 0.12)	0.12 (0.01, 0.23)	0.02 (-0.22, 0.19)	0.03 (-0.01, 0.08)
Sex, male versus female (ref)	-2.85 (-4.95, -0.75)	-5.36 (-10.04, -0.68)	0.46 (-5.71, 6.62)	-0.65 (-2.43, 1.13)
PO2/FIO2 ratio, one-unit increment	-0.01 (-0.02, -0.01)	-0.02 (-0.03, 0.01)	-0.02 (-0.06, 0.02)	-0.01 (-0.01, 0.01)
APACHE II, one-unit increment	0.01 (-0.11, 0.14)	0.15 (-0.06, 0.35)	-0.01 (-0.50, 0.48)	-0.03 (-0.16, 0.09)
SOFA score, one-unit increment	0.17 (-0.14, 0.48)	-0.10 (-0.59, 0.40)	0.66 (-0.34, 1.66)	0.39 (0.03, 0.75)
Hypertension, yes versus no (ref)	0.02 (-1.49, 1.54)	0.16 (-2.66, 2.98)	0.66 (-4.44, 5.76)	0.35 (-0.93, 1.63)
Diabetes, yes versus no (ref)	-1.08 (-2.46, 0.30)	-1.88 (-.4.57, 0.81)	-2.13 (-6.85, 2.59)	0.06 (-1.06, 1.17)
Kidney disease, yes versus no (ref)	2.73 (-0.40, 5.85)	0.14 (-5.62, 5.89)	8.59 (-0.82, 17.99)	-1.13 (-4.10, 1.83)
Kidney disease on dialysis, yes versus no (ref)	-1.96 (-7.46, 3.55)	-11.94 (-23.92, 0.04)	-2.19 (-26.47, 22.09)	1.29 (-2.66, 5.24)
Liver disease, yes versus no (ref)	1.34 (-8.04, 10.73)	2.52 (-21.66, 26.70)	-6.42 (-28.47, 15.63)	-4.04 (-13.85, 5.78)
Heart disease, yes versus no (ref)	-0.52 (-2.76, 1.73)	-1.65 (-5.98, 2.68)	-0.08 (-6.99, 6.84)	-0.98 (-3.01, 1.06)
Lung disease, yes versus no (ref)	-0.61 (-3.52, 2.30)	-2.24 (-8.19, 3.70)	2.17 (-6.79, 11.13)	0.18 (-2.21, 2.58)
Immunosuppression, yes versus no (ref)	-2.82 (-7.45, 1.80)	-0.53 (-11.04, 9.98)	-5.19 (-18.26, 7.87)	-2.68 (-6.68, 1.31)
Metastatic cancer, yes versus no (ref)	0.06 (-6.26, 6.38)	-5.67 (-18.86, 7.51)	-6.06 (-31.59, 19.46)	4.68 (-0.48, 9.83)
Hematological malignancy, yes versus no (ref)	-2.41 (-8.97, 4.15)	2.22 (-13.36, 17.79)	-5.82 (-18.82, 7.18)	-^a^

Further, intubation was associated with higher ICU days in the total group (Beta = 6.78, 95% CI: 5.35, 8.21). Increased age was associated with higher ICU days, while the male sex was associated with lower ICU stay days in the total and early intubated groups (Table 5). Interestingly, an increased PO2/FIO2 ratio was associated with lower ICU stay days in the total group (Beta = -0.01, 95% CI: -0.02, -0.01), while an increased SOFA score was associated with higher ICU stay days in the not intubated group (Beta = 0.39, 95% CI: 0.03, 0.75). Further, we saw similar associations between intubation, increased age, male sex, increased PO2/FIO2 ratio, and SOFA score in the subsample of alive patients (Table 6).

**Table 6:** Factors associated with ICU stay days of alive patients There were 49, 3, 7, and 39 missing values in total, early, late, and not intubated groups. Models were constructed using generalized linear regression with the ICU stay days as the continuous outcome. Betas represent an increase or decrease in the ICU stay days for every unit increase in continuous variables, and intubation, male sex, the presence of comorbidity compared to the not intubated, female sex, and absence of comorbidity. ^a^Exposures with 0 values in some groups. CI: confidence interval; N/A: not applicable; ref: reference category; ICU: intensive care unit; APACHE II: Acute Physiology and Chronic Health Evaluation II; SOFA: Sequential Organ Failure Assessment

Patient characteristics	Total (n = 543)	Early intubated (n = 181)	Late intubated (n = 70)	Not intubated (n = 292)
	Beta (95% CI)	Beta (95% CI)	Beta (95% CI)	Beta (95% CI)
Intubation, yes versus no (ref)	6.31 (5.05, 7.57)	N/A	N/A	N/A
Age (years), one-year increment	0.05 (0.01, 0.11)	0.10 (-0.01, 0.22)	0.08 (-0.13, 0.30)	0.03 (-0.01, 0.08)
Sex, male versus female (ref)	-2.72 (-4.70, -0.74)	-4.96 (-9.60, -0.32)	-5.87 (-13.39, 1.66)	-0.65 (-2.44, 1.13)
PO2/FIO2 ratio, one-unit increment	-0.01 (-0.02, -0.01)	-0.02 (-0.03, 0.01)	-0.01 (-0.05, 0.05)	-0.01 (-0.01, 0.01)
APACHE II, one-unit increment	0.05 (-0.07, 0.16)	0.17 (-0.05, 0.39)	-0.17 (-0.76, 0.43)	-0.03 (-0.16, 0.09)
SOFA score, one-unit increment	0.31 (0.01, 0.61)	0.24 (-0.28, 0.76)	0.36 (-0.78, 1.50)	0.39 (0.03, 0.75)
Hypertension, yes versus no (ref)	0.58 (-0.81, 1.97)	0.16 (-2.75, 3.06)	4.53 (-1.08, 10.14)	0.35 (-0.93, 1.63)
Diabetes, yes versus no (ref)	-0.53 (-1.80, 0.73)	-1.72 (-.4.47, 1.03)	-0.10 (-5.21, 5.01)	0.06 (-1.06, 1.17)
Kidney disease, yes versus no (ref)	0.37 (-2.75, 3.49)	-0.07 (-6.11, 5.98)	-3.19 (-18.32, 11.95)	-1.13 (-4.10, 1.83)
Kidney disease on dialysis, yes versus no (ref)	-0.80 (-5.86, 4.28)	-5.68 (-21.79, 10.42)	-15.37 (-39.70, 8.95)	1.29 (-2.66, 5.24)
Liver disease, yes versus no (ref)	-6.37 (-21.08, 8.34)	-^a^	-^a^	-4.04 (-13.86, 5.79)
Heart disease, yes versus no (ref)	-1.22 (-3.44, 1.00)	-2.50 (-6.95, 1.95)	8.20 (-3.93, 20.33)	-0.98 (-3.01, 1.06)
Lung disease, yes versus no (ref)	-0.97 (-3.65, 1.71)	-1.84 (-7.46, 3.78)	1.62 (-10.37, 13.61)	0.18 (-2.21, 2.58)
Immunosuppression, yes versus no (ref)	-4.72 (-9.69, 0.25)	-9.45 (-24.80, 5.89)	-1.72 (-19.87, 16.43)	-2.68 (-6.68, 1.31)
Metastatic cancer, yes versus no (ref)	5.86 (-1.58, 13.30)	-^a^	-^a^	4.68 (-0.48, 9.84)
Hematological malignancy, yes versus no (ref)	-3.51 (-16.14, 9.12)	-^a^	-3.06 (-20.82, 14.70)	-^a^

In the model that included both dead and alive patients, hospital mortality was associated with lower hospital stay days in the total, early, and not intubated groups (Beta = -4.05, 95% CI: -6.66, -1.43; Beta = -5.72, 95% CI: -9.80, -1.64; and Beta = -25.72, 95% CI: -40.28, -11.17, respectively), while intubation was associated with higher hospital stay days in the total group (Beta = 6.27, 95% CI: 4.46, 8.08). Increased age was associated with higher hospital stay days in the total (Beta = 0.13, 95% CI: 0.06, 0.20) and not intubated (Beta = 0.18, 95% CI: 0.10, 0.26) groups, while the male sex was associated with lower hospital stay days in the early intubated group (Beta = -8.20, 95% CI: -14.94, -1.46). Interestingly, kidney disease on dialysis was associated with higher hospital stay days in the total and not intubated groups, and metastatic cancer was associated with higher hospital stay days in the not intubated group. The presence of hypertension was associated with higher hospital stay days in the early intubated group (Table 7).

**Table 7:** Factors associated with hospital stay days of patients (dead or alive) There were 103, 35, 24, and 43 missing values in the total, early, late, and not intubated groups. Models were constructed using generalized linear regression with the hospital stay days as the continuous outcome. Betas represent an increase or decrease in the hospital stay days for every unit increase in continuous variables, and hospital mortality, intubation, male sex, the presence of comorbidity compared to the hospital alive, not intubated, female sex, and absence of comorbidity. ^a^Exposures with 0 values in some groups. CI: confidence interval; N/A: not applicable; ref: reference category; APACHE II: Acute Physiology and Chronic Health Evaluation II; SOFA: Sequential Organ Failure Assessment

Patient characteristics	Total (n = 583)	Early intubated (n = 187)	Late intubated (n = 107)	Not intubated (n = 289)
	Beta (95% CI)	Beta (95% CI)	Beta (95% CI)	Beta (95% CI)
Hospital mortality, yes versus no (ref)	-4.05 (-6.66, -1.43)	-5.72 (-9.80, -1.64)	-2.30 (-8.06, 3.46)	-25.72 (-40.28, -11.17)
Intubation, yes versus no (ref)	6.27 (4.46, 8.08)	N/A	N/A	N/A
Age (years), one-year increment	0.13 (0.06, 0.20)	0.02 (-0.12, 0.15)	0.11 (-0.11, 0.32)	0.18 (0.10, 0.26)
Sex, male versus female (ref)	-2.10 (-4.81, 0.62)	-8.20 (-14.94, -1.46)	2.25 (-4.31, 8.81)	0.57 (-2.45, 3.59)
PO2/FIO2 ratio, one-unit increment	-0.01 (-0.02, 0.01)	-0.01 (-0.04, 0.01)	-0.02 (-0.06, 0.02)	0.01 (-0.01, 0.01)
APACHE II, one-unit increment	-0.08 (-0.24, 0.08)	0.09 (-0.16, 0.34)	-0.22 (-0.74, 0.30)	-0.08 (-0.30, 0.13)
SOFA score, one-unit increment	0.06 (-0.35, 0.46)	-0.49 (-1.09, 0.12)	1.03 (-0.01, 2.06)	0.52 (-0.12, 1.15)
Hypertension, yes versus no (ref)	0.63 (-1.26, 2.52)	3.70 (0.36, 7.03)	-2.30 (-7.78, 3.18)	0.14 (-2.04, 2.32)
Diabetes, yes versus no (ref)	-1.72 (-3.46, 0.01)	-1.74 (-4.97, 1.50)	-2.17 (-7.26, 2.92)	-1.31 (-3.22, 0.60)
Kidney disease, yes versus no (ref)	3.66 (-0.55, 7.87)	-3.30 (-11.02, 4.41)	10.65 (-0.33, 21.62)	4.12 (-1.15, 9.38)
Kidney disease on dialysis, yes versus no (ref)	9.60 (2.48, 16.71)	5.04 (-8.62, 18.71)	-^a^	9.84 (3.14, 16.53)
Liver disease, yes versus no (ref)	5.28 (-6.15, 16.71)	-0.76 (-29.08, 27.55)	-4.22 (-26.94, 18.50)	-1.28 (-17.92, 15.37)
Heart disease, yes versus no (ref)	-1.12 (-3.96, 1.74)	1.09 (-4.09, 6.28)	-1.67 (-9.14. 5.80)	-2.21 (-5.68, 1.26)
Lung disease, yes versus no (ref)	2.78 (-0.89, 6.45)	3.93 (-3.49, 5.81)	6.40 (-3.24, 16.03)	1.31 (-2.77, 5.40)
Immunosuppression, yes versus no (ref)	-2.94 (-8.57, 2.69)	-3.20 (-15.23, 8.84)	-4.66 (-18.08, 8.77)	-1.61 (-8.39, 5.16)
Metastatic cancer, yes versus no (ref)	6.96 (-0.74, 14.65)	-3.44 (-18.67, 11.78)	-0.49 (-26.77, 25.78)	14.38 (5.63, 23.14)
Hematological malignancy, yes versus no (ref)	1.65 (-6.32, 9.62)	11.74 (-6.06, 29.53)	-3.61 (-17.08, 9.85)	-^a^

Further, we observed similar associations of intubation, increased age, male sex, kidney disease on dialysis, and metastatic cancer with hospital stay days among alive patients (Table 8).

**Table 8:** Factors associated with hospital stay days of alive patients ^a^Exposures with 0 values in some groups. CI: confidence interval; N/A: not applicable; ref: reference category; APACHE II: Acute Physiology and Chronic Health Evaluation II; SOFA: Sequential Organ Failure Assessment

Patient characteristics	Total (n = 503)	Early intubated (n = 155)	Late intubated (n = 60)	Not intubated (n = 288)
	Beta (95% CI)	Beta (95% CI)	Beta (95% CI)	Beta (95% CI)
Intubation, yes versus no (ref)	5.87 (4.19, 7.55)	N/A	N/A	N/A
Age (years), one-year increment	0.15 (0.08, 0.22)	0.05 (-0.09, 0.20)	0.19 (-0.06, 0.44)	0.18 (0.10, 0.26)
Sex, male versus female (ref)	-2.13 (-4.86, 0.60)	-8.64 (-15.26, -2.03)	-4.10 (12.83, 4.62)	0.57 (-2.46, 3.59)
PO2/FIO2 ratio, one-unit increment	-0.01 (-0.02, 0.01)	-0.02 (-0.04, 0.01)	-0.01 (-0.06, 0.05)	0.01 (-0.01, 0.01)
APACHE II, one-unit increment	-0.07 (-0.24, 0.09)	0.08 (-0.18, 0.33)	-0.60 (-1.31, 0.12)	-0.08 (-0.30, 0.13)
SOFA score, one-unit increment	0.19 (-0.22, 0.60)	-0.02 (-0.63, 0.60)	0.68 (-0.61, 1.96)	0.52 (-0.12, 1.15)
Hypertension, yes versus no (ref)	1.41 (-0.41, 3.24)	4.42 (1.12, 7.72)	1.22 (-5.27, 7.72)	0.14 (-2.04, 2.32)
Diabetes, yes versus no (ref)	-1.43 (-3.09, 0.24)	-1.84 (-5.04, 1.34)	-0.31 (-6.14, 5.53)	-1.31 (-3.23, 0.60)
Kidney disease, yes versus no (ref)	0.01 (-4.62, 4.65)	-4.41 (-12.49, 3.66)	-17.22 (-44.36, 9.93)	4.12 (-1.16, 9.39)
Kidney disease on dialysis, yes versus no (ref)	12.53 (5.58, 19.48)	26.56 (8.97, 44.15)	-^a^	9.84 (3.13, 16.54)
Liver disease, yes versus no (ref)	-1.85 (-20.66, 16.96)	-^a^	-^a^	-1.28 (-17.95, 15.40)
Heart disease, yes versus no (ref)	-2.02 (-5.01, 0.97)	0.28 (-4.74, 5.30)	-8.35 (-27.98. 11.27)	-2.21 (-5.69, 1.27)
Lung disease, yes versus no (ref)	2.93 (-0.65, 6.51)	4.71 (-2.03, 11.44)	10.80 (-3.41, 25.01)	1.31 (-2.78, 5.40)
Immunosuppression, yes versus no (ref)	-4.40 (-10.77, 1.97)	-11.08 (-27.79, 5.63)	-3.41 (-22.93, 16.11)	-1.61 (-8.40, 5.18)
Metastatic cancer, yes versus no (ref)	14.32 (4.78, 23.85)	-^a^	-^a^	14.38 (5.62, 23.15)
Hematological malignancy, yes versus no (ref)	-0.84 (-16.98, 15.29)	-^a^	4.59 (-23.58, 14.39)	-^a^

Additionally, among the alive patients, kidney disease on dialysis was associated with higher hospital stay days in the early intubated groups. In comparison, metastatic cancer was associated with higher hospital stay days in the whole group.

## Discussion

As seen in the results provided, early intubation was not associated with higher mortality when compared to a late intubation strategy, while the best outcome was seen in the non-intubated group. The early intubation group fared better in comparison to the late intubation group. In comparison to an earlier meta-analysis, for which a total of 12 studies included 8,944 critically ill patients with COVID-19 and where the early intubation group was defined as intubation within 24 hours of hospital admission, Papoutsi et al suggested that the timing of intubation may have no effect on mortality and morbidity of critically ill patients with COVID-19 [8]; this may in part be due to the earlier implementation of protective lung ventilation strategies, in contrast to increased work of breathing, the incidence of patient self-inflicted lung injury (P-SILI), and lung parenchymal damage from high FIO2 requirements in the latter group. Spontaneous breathing patient with hypoxic respiratory failure has a high respiratory drive, which, in turn, leads to swinging transpulmonary pressure, and is associated with increased pulmonary vascular leakage; these phenomena (patient self-inflicted lung injury (P-SILII)) result in more insult to the already injured lung in a SARS-CoV-2 ARDS patient. Brochard et al. have suggested that the implication of a lung-protective ventilation strategy would be feasible and protective against P-SILI [11]. The worst outcome of the late intubation group could be also related to the low average PO2/FIO2 ratio in that group. Additionally, those patients were more at risk of self-inflicting lung injury (P-SILI) with NIPPV, in which the tidal volume, respiratory drive, and mechanical power [12] would be a noncontrolled variable and can lead to further deterioration of the lung injury [13].

Interestingly, our data has also shown that patients in the early intubation group were clinically more severe; they presented with higher APACHE II and SOFA scores compared to the other groups, which can be attributed to the intense inflammatory response associated with the SARS-CoV-2 infection. Stratifying patients with their comorbidities revealed that the elevated risk groups include the older age group (>60) and patients with hypertension (HTN), diabetes mellitus (DM), chronic kidney disease (CKD), chronic liver disease (CLD), and malignancies. High APACHE II and SOFA scores and low PO2/FIO2 ratios were independent risk factors for higher mortality in the ICU and hospital settings. The time of intubation in ARDS patients in general and COVID-19 ARDS is still a matter of debate. A constellation of clinical, physiological, and laboratory criteria can help predict the time of intubation and the need for transition from noninvasive to invasive ventilation [14], e.g., the respiratory rate-oxygenation (ROX) index, which can help predict a successful HFNC ventilation strategy [15]. Another predictor that has been proposed by Slutsky et al., which depends on several points, including age, vasopressor use, comorbidities, Glasgow Coma Scale (GCS), and oxygen index, has been in place, which could help categorize patients into high- and low-risk groups regarding the initiation and continuation of NIV [16]. The current standard care for patients with COVID-19 ARDS has not been different from the usual care for non-COVID-19 ARDS, which includes the ventilatory management of low tidal volume, prone ventilation, positive end-expiratory pressure (PEEP) titration, the use of pharmacotherapy such as muscle relaxant, and the utilization of extracorporeal oxygenation in the severe form. Additionally, in COVID-19 ARDS, the initiation of dexamethasone has been shown to reduce mortality [17]. Unfortunately, this approach would not be possible for a spontaneous breathing patient to whom the concept of low tidal ventilation and muscle relaxant administration would not be applicable. Another point to consider in our study is that patients who required late intubation failed treatment response and did not do well on NIV initially, which could explain the worse outcome in the late intubation group.

The limitation of our study is that it is a retrospective, observational study, and it was conducted in a single tertiary center, which would limit the concept of generalizability, although the nature of our patient from a different geographical background would count positively toward the generalizability. Another factor that would count for the mortality difference is that early during the COVID-19 epidemic, the trend was toward early intubation to minimize the viral particle dispersion, which would explain the higher average PO2/FIO2 ratios in the early intubation arm.

## Conclusions

In our study, COVID-19 ARDS patients who required early invasive ventilatory support and in whom the physiological parameter was more severe (APACHE II and SOFA scores) had a better outcome than the late intubation group. Age more than 60 years, diabetes, hypertension, CKD, and CLD were the main predictor of mortality in total.
